# Maintenance of neuronal size gradient in MNTB requires sound-evoked activity

**DOI:** 10.1152/jn.00528.2016

**Published:** 2016-11-23

**Authors:** Jessica H. Weatherstone, Conny Kopp-Scheinpflug, Nadia Pilati, Yuan Wang, Ian D. Forsythe, Edwin W. Rubel, Bruce L. Tempel

**Affiliations:** ^1^Virginia Merrill Bloedel Hearing Research Center, Department of Otolaryngology-Head and Neck Surgery, and Department of Pharmacology, University of Washington School of Medicine, Seattle, Washington;; ^2^Virginia Merrill Bloedel Hearing Research Center, Department of Otolaryngology-Head and Neck Surgery, and Department of Physiology and Biophysics, University of Washington School of Medicine, Seattle, Washington;; ^3^Department of Neuroscience, Psychology and Behaviour, University of Leicester, Leicester, United Kingdom;; ^4^Division of Neurobiology, Department Biology II, Ludwig-Maximilians University Munich, Planegg-Martinsried, Germany;; ^5^Autifony Srl Laboratories, Medicines Research Centre, Verona, Italy; and; ^6^Department of Biomedical Sciences, College of Medicine, Florida State University, Tallahassee, Florida

**Keywords:** PMCA2, calyx of Held, synaptic transmission, auditory brain stem, tonotopic gradients

## Abstract

The medial nucleus of the trapezoid body (MNTB) is an important source of inhibition during the computation of sound location. It transmits fast and precisely timed action potentials at high frequencies; this requires an efficient calcium clearance mechanism, in which plasma membrane calcium ATPase 2 (PMCA2) is a key component. Deafwaddler (*dfw*^*2J*^) mutant mice have a null mutation in PMCA2 causing deafness in homozygotes (*dfw*^*2J*^/*dfw*^*2J*^) and high-frequency hearing loss in heterozygotes (+/*dfw*^*2J*^). Despite the deafness phenotype, no significant differences in MNTB volume or cell number were observed in *dfw*^*2J*^ homozygous mutants, suggesting that PMCA2 is not required for MNTB neuron survival. The MNTB tonotopic axis encodes high to low sound frequencies across the medial to lateral dimension. We discovered a cell size gradient along this axis: lateral neuronal somata are significantly larger than medially located somata. This size gradient is decreased in +/*dfw*^*2J*^ and absent in *dfw*^*2J*^/*dfw*^*2J*^. The lack of acoustically driven input suggests that sound-evoked activity is required for maintenance of the cell size gradient. This hypothesis was corroborated by selective elimination of auditory hair cell activity with either hair cell elimination in Pou4f3 DTR mice or inner ear tetrodotoxin (TTX) treatment. The change in soma size was reversible and recovered within 7 days of TTX treatment, suggesting that regulation of the gradient is dependent on synaptic activity and that these changes are plastic rather than permanent.

**NEW & NOTEWORTHY** Neurons of the medial nucleus of the trapezoid body (MNTB) act as fast-spiking inhibitory interneurons within the auditory brain stem. The MNTB is topographically organized, with low sound frequencies encoded laterally and high frequencies medially. We discovered a cell size gradient along this axis: lateral neurons are larger than medial neurons. The absence of this gradient in deaf mice lacking plasma membrane calcium ATPase 2 suggests an activity-dependent, calcium-mediated mechanism that controls neuronal soma size.

## Abstract (precis)

Neurons of the medial nucleus of the trapezoid body (MNTB) act as fast-spiking inhibitory interneurons within the auditory brain stem. The MNTB is topographically organized, with low sound frequencies encoded laterally and high frequencies medially. We discovered a cell size gradient along this axis: lateral neurons are larger than medial neurons. The absence of this gradient in deaf mice lacking plasma membrane calcium ATPase 2 suggests an activity-dependent, calcium-mediated mechanism that controls neuronal soma size.

action potentials generated from both ears are transmitted to the superior olivary complex via the globular and spherical bushy cells of the anterior ventral cochlear nucleus. Ipsilateral excitatory and contralateral inhibitory projections are integrated in the lateral superior olive (LSO) to calculate interaural intensity differences (see Tollin 2003 for review). Although the excitatory input to the LSO is direct, the inhibitory circuit includes a signal inversion upon transmission through the medial nucleus of the trapezoid body (MNTB). These projections must converge in temporal register (Tollin 2003) and hence require fast transmission in the globular bushy cell-MNTB pathway to compensate for the additional synapse (Taschenberger and von Gersdorff 2000; Wang et al. 1998). MNTB neurons are driven by large glutamatergic synapses, the calyces of Held (Schneggenburger and Forsythe 2006; von Gersdorff and Borst 2002), and can sustain in vivo instantaneous firing rates of >300 spikes/s (Kopp-Scheinpflug et al. 2008). With such high firing frequencies, presynaptic residual calcium must be cleared rapidly to avoid synaptic facilitation and/or depression. Similarly, calcium accumulation must also be controlled in the postsynaptic MNTB neuron.

PMCA2, the most efficient of the plasma membrane calcium ATPases, is localized in the stereocilia of sensory hair cells in the cochlea and is necessary for hair cell survival (Dumont et al. 2001; Kozel et al. 1998, 2002; McCullough and Tempel 2004; Street et al. 1998; Takahashi and Kitamura 1999; Yamoah et al. 1998). Spontaneous mutations in the gene that encodes PMCA2 decrease expression and are associated with hearing loss in both humans and mice (Brini et al. 2007; Ficarella et al. 2007; McCullough et al. 2007; Schultz et al. 2005). These mutations in mice provide a valuable genetic tool to study PMCA2 in a mammalian model. The first PMCA2 mutant discovered was *deafwaddler* (*dfw*), which results in a phenotype with auditory and vestibular deficits. The *dfw* point mutation renders the PMCA2 pump 60% less efficient compared with the wild type (WT) (Penheiter et al. 2001; Street et al. 1998). Another example is the *dfw*^*2J*^ mutation, which is a frameshift mutation resulting in a premature stop codon (Street et al. 1998). Homozygous *dfw*^*2J*^ mutants (*dfw*^*2J*^/*dfw*^*2J*^) produce no PMCA2 protein, causing a more severe phenotype of deafness and ataxia, while heterozygous mutants (+/*dfw*^*2J*^) exhibit a phenotype limited to high-frequency hearing loss (McCullough et al. 2007). PMCA2 is highly expressed in avian brain stem neurons involved in sound localization, and its expression is regulated by synaptic activity (Wang et al. 2009), but little is known about PMCA2 expression and function in the central auditory pathway of mammals.

Here we used anatomical, pharmacological, and electrophysiological methods to study the expression and function of PMCA2 in the MNTB. We show that, unlike in the peripheral auditory system, PMCA2 is not necessary for neuronal survival in the MNTB. Unexpectedly, we discovered a tonotopically organized cell size gradient in the MNTB that is regulated by sound-evoked activity and is absent in deaf PMCA2 mutants.

## MATERIALS AND METHODS

### 

#### Animals.

Adult (5–7 wk old) CBA/CaJ deafwaddler (*dfw*^*2J*^), CBA/CaJ deafwaddler (*dfw*) (Street et al. 1998), and Pou4f3 DTR (Golub et al. 2012; Mahrt et al. 2013; Tong et al. 2015) mice of either sex were obtained from the University of Washington breeding colonies. Mice were genotyped with DNA obtained from tail biopsies. PCR amplification of the mutation (dfw) or insertion [diphtheria toxin receptor (DTR)] were electrophoresed through an agarose gel, and samples were detected with ethidium bromide and a transilluminator. For *dfw*^*2J*^ mutants, genotyping was done with a TaqMan SNP genotyping assay (Applied Biosciences). Detailed protocols are available online (http://depts.washington.edu/tempelab/Protocols/DFW2J.html). All manipulations were carried out in accordance with protocols approved by the University of Washington Animal Care Committee and were performed in accordance with the National Institutes of Health *Guide for the Care and Use of Laboratory Animals*.

#### Diphtheria toxin treatment.

Diphtheria toxin (DT) was administered to DTR mice, genetically engineered to express the human DTR selectively in hair cells (Tong et al. 2015). A single 25 μg/kg dose of DT (List Biological Laboratories, no. 150) was delivered via intramuscular injection to 4-wk-old DTR mice. Within 6 days after DT injection, DTR mice lose all of their hair cells and are completely deaf (Tong et al. 2015). After DT injection, DTR mice were allowed to survive for 2 wk before tissue collection.

#### Histology.

The animals were anesthetized with an overdose of Nembutal and perfused with a saline-heparin solution followed by 4% paraformaldahyde. The brains were exposed in the skull and stored in 4% paraformaldahyde overnight. The brains were then dissected from the skull and postfixed for an additional hour. The tissue was transferred to 10% sucrose in 0.1 M phosphate buffer until sinking, which took ∼3 h. The tissue was transferred again to 30% sucrose in 0.1 M phosphate buffer, where it remained until sinking, which took ∼24 h. Coronal sections of 10- or 40-μm thickness were cut through the brain stem with a cryostat or freezing stage on a sledge microtome. Free-floating sections were stored in phosphate-buffered saline (PBS; pH 7.4).

#### Immunocytochemistry.

The fixed sections were treated with primary antibody for PMCA2 (dilution 1:250) in PBS with 0.3% Triton X-100 for 2 h at room temperature and washed in PBS overnight at 4°C. The sections were then incubated with microtubule-associated protein 2 (MAP2) primary antibody at 1:1,000 in PBS with 0.3% Triton X-100 for 1.5 h at room temperature. The sections were washed in PBS before incubation in Alexa Fluor secondary antibodies (1:200; Molecular Probes, Eugene, OR) for 2 h at room temperature. The sections were treated with DAPI before being coverslipped with Fluoromount-G (Southern Biotech).

#### Primary antibodies.

Polyclonal anti-PMCA2 (catalog no. PA1-915, rabbit) was purchased from Affinity Bioreagents (Golden, CO). The immunogen was a synthetic peptide corresponding to amino acid residues 5–19 of human PMCA2 protein, sequence: TNSDFYSKNQRNESS. This sequence is conserved between human and mouse PMCA2. Monoclonal anti-MAP2 (catalog no. MAB3418, mouse) was purchased from Chemicon International. The immunogen was bovine brain microtubule protein and binds to MAP2a and MAP2b.

#### Nissl staining.

Alternate sections from each animal were mounted and stained with thionine for 5 min and then dehydrated in xylene, mounted, and coverslipped with DPX (Sigma).

#### Confocal microscopy.

Images for the immunocytochemistry experiment were taken with an Olympus FV-1000 confocal microscope with an oil ×100 objective. A 5.6-μm-thick z stack was deconvolved with the Huygens deconvolution system. The image was cropped to contain one cell (∼1/4th of original image). Brightness and contrast were adjusted to maximize visualization of the calyx.

#### Light microscopy.

Images for morphology experiments were taken with a Zeiss Axioplan 2ie using a ×10 or ×40 objective. Each section was positioned so that the midline was perpendicular or parallel to the *x*-axis of the image. The focal plane selected for these images was approximately in the center of the section thickness to the nearest micrometer. For ×10 magnification one image was taken. For ×40 magnification 1–16 images were taken covering the entirety of the MNTB in that section. The ×40 images were used to generate a montage with MosaicJ in ImageJ and saved as one image.

#### Profile counts.

To determine the number of neurons in each MNTB, all neurons in the MNTB of stained sections were counted online with a counting grid. The slides were randomized to blind the counter to the genotype of the tissue. The experimenter focused up and down with a ×40 objective in each square of the counting grid. Only neurons with a nucleus and a nucleolus were counted. The total number of neurons present in each MNTB was estimated by multiplying by 2, since only half of the slices were analyzed (see Fig. 2*A*).

#### MNTB volume.

The volume of the nucleus was determined with the cross-sectional area of the MNTB in each thionine-stained section. Images of the MNTB in each section were taken with a ×10 lens and randomized for blind analysis. The MNTB was outlined with ImageJ; only cells that were darkly stained and <20 μm from their nearest neighbor were included in the MNTB perimeter. This outline was used to calculate the area of the MNTB in each section. The volume of the MNTB was estimated by multiplying each MNTB area by 40 μm. This value was doubled, since only every other section of the MNTB was analyzed. These individual areas were summed to find the total volume of each MNTB (see Fig. 2*B*).

#### Neuron size.

Neuron size was measured with ×40 montaged images of coronal sections such that the montage included the entire extent of the nucleus in any given section. Each montaged image was given a random number file name to blind the experimenter to genotype and subject identity. All cells in the montaged image that contained a defined nucleus, nucleolus, and unobstructed cell membrane were analyzed. The cross-sectional area of the neuron as well as the *x*- and *y*-coordinates of the region of interest's central pixel within the image were obtained with the algorithm provided by ImageJ (Fig. 2*C*). The *x*- and *y*-coordinates were then used to calculate the distance from the midline of the brain section for each individual neuron.

#### Tonotopic axis.

The tonotopic gradient in the MNTB extends from neurons encoding high frequencies dorsomedially to neurons encoding low frequencies ventrolaterally (Sonntag et al. 2009). Therefore the tonotopic axis was defined as the longest dorsomedial-to-ventrolateral line that could be drawn through the MNTB to estimate the expected tonotopic axis in each montaged image of the coronal sections. This line was divided into thirds, and then two additional lines were drawn perpendicular to the tonotopic axis to delineate medial, central, and lateral areas.

#### Slice preparations.

Mice (postnatal days 13–20) were killed by decapitation in accordance with the UK Animals (Scientific Procedures) Act 1986, and brain stem slices containing the superior olivary complex were prepared as previously described (Tong et al. 2013). Transverse slices (200 μm thick) containing the MNTB were cut in a low-sodium artificial CSF (aCSF) at ∼0°C. Slices were maintained in a normal aCSF at 37°C for 1 h, after which they were stored at room temperature (∼20°C) in a continually recycling slice maintenance chamber. Composition of the normal aCSF was (mM) 125 NaCl, 2.5 KCl, 26 NaHCO_3_, 10 glucose, 1.25 NaH_2_PO_4_, 2 sodium pyruvate, 3 *myo*-inositol, 2 CaCl_2_, 1 MgCl_2_, and 0.5 ascorbic acid; pH was 7.4, bubbled with 95% O_2_-5% CO_2_. For the low-sodium aCSF, NaCl was replaced by 250 mM sucrose and CaCl_2_ and MgCl_2_ concentrations were changed to 0.1 and 4 mM, respectively. Experiments were conducted at a temperature of 36 ± 1°C with a Peltier-driven environmental chamber (constructed by University of Leicester Mechanical and Electronic Joint Workshops) or with a CI7800 (Campden Instruments) feedback temperature controller.

#### Patch-clamp recording.

Whole cell patch-clamp recordings were made from visually identified MNTB neurons (×40 water-immersion objective, differential interference contrast optics) with an Axopatch 200B amplifier/Digidata 1440 (synaptic physiology) or a Multiclamp 700B amplifier (capacitance measures) and pCLAMP 10 software (Molecular Devices, Sunnyvale, CA), sampling at 50 kHz and filtering at 10 kHz. Patch pipettes were pulled from borosilicate glass capillaries (GC150F-7.5, OD: 1.5 mm; Harvard Apparatus, Edenbridge, UK) with a two-stage vertical puller (PC-10; Narishige, Tokyo, Japan). Their resistance was ∼3.0 MΩ when filled with a patch solution containing (mM) 97.5 K gluconate, 32.5 KCl, 40 HEPES, 5 EGTA, 1 MgCl_2_, and 5 Na_2_ phosphocreatine; pH was adjusted to 7.2 with KOH. Osmolarity was ∼300 mosM. Voltage signals were not corrected for the liquid junction potential (−11 mV). Whole cell series resistances were <10 MΩ, compensated by 70%, and recordings in which the series resistance changed >2 MΩ were eliminated from analysis. Excitatory postsynaptic currents (EPSCs) were elicited by stimulation through a bipolar platinum electrode positioned across the midline. The stimulating electrode was connected to a voltage stimulator (DS2A; Digitimer) delivering 200-μs, 5- to 50-V pulses at a rate of 0.25 Hz. The voltage stimulus was adjusted to give a large synaptic response from one calyceal input in each recording. EPSCs were recorded in the presence of 10 μM bicuculline, 0.5–1 μM strychnine, and 50 μM 2-amino-5-phosphonopentanoic acid (d-AP5). Tetrodotoxin (TTX; 0.5 μM) was added in addition to the above cocktail to record miniature EPSCs (mEPSCs). All chemicals and drugs were obtained from Sigma UK, with the exception of bicuculline and d-AP5 from Tocris (Bristol, UK). EPSC decay times and amplitudes were measured from averaged traces (10–15 records). mEPSC decay times were measured from averaged traces (20 records). The holding potential was set to −40 mV.

#### Capacitance measures.

Cell capacitance was assessed in whole cell voltage-clamp recordings with pCLAMP 10 software. For each neuron the capacitance value was read out directly as the telegraphed signal from the amplifier. At the end of each recording, a low-magnification (×4) image was taken to document the location of the pipette tip (still in the cell) with respect to the midline. These images were then used to divide the MNTB into medial, central, and lateral divisions as introduced above.

#### In vivo recordings.

**Fig. 1.**
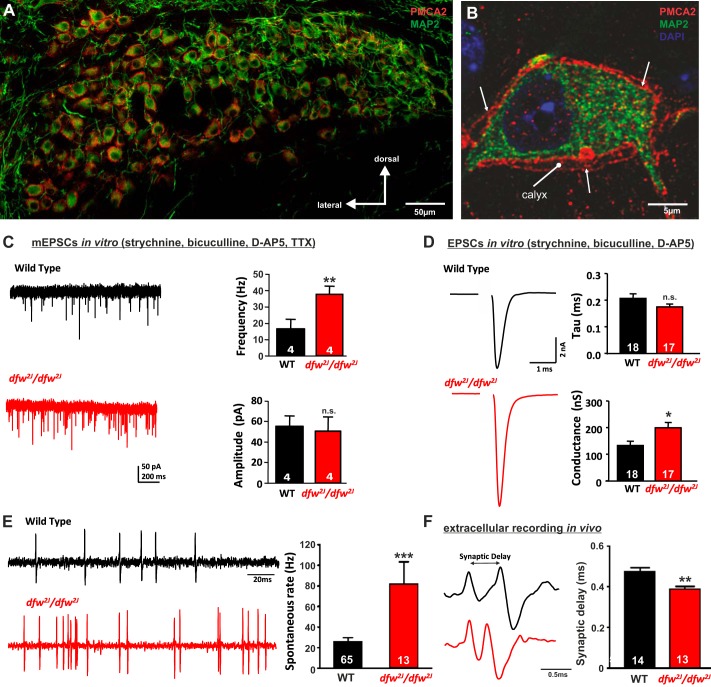
PMCA2 regulates transmitter release in the MNTB. *A* and *B*: immunohistochemical labeling for MAP2 and PMCA2 in the MNTB (*A*) and in an individual MNTB neuron (*B*). A cross section through the calyx is marked “calyx.” Arrows show where PMCA2 appears to be localized presynaptically in the outer membrane of the calyx. *C*: voltage-clamp recordings from postsynaptic MNTB neurons in acute brain slices show a higher frequency of miniature excitatory postsynaptic currents (mEPSCs) in the MNTB of *dfw*^*2J*^/*dfw*^*2J*^ mice compared with wild type (WT). *D*: calyceal EPSCs evoked by midline stimulation are larger in *dfw*^*2J*^/*dfw*^*2J*^ mice compared with WT. Stimulus artifacts have been deleted for clarity. WT data include 7 medial cells, 3 lateral cells, and 8 cells with no information about location in the MNTB. *dfw*^*2J*^/*dfw*^*2J*^ data include 7 medial cells, 6 lateral cells, and 4 cells with no information about location in the MNTB (see also Fig. 4*C*). *E* and *F*: in vivo single-unit recordings of MNTB neurons measured higher spontaneous firing rates (*E*) and shorter synaptic delays (*F*) in *dfw*^*2J*^/*dfw*^*2J*^ mice compared with WT. ***P ≤ 0.001, **P ≤ 0.01, *P ≤ 0.05, n.s., Not significant.

Spontaneous and sound-evoked MNTB neuron responses were recorded from 16 adult mice (3 *dfw*^*2J*^/*dfw*^*2J*^, 13 WT CBA/Ca). During surgical preparation and recording, animals were anesthetized by intraperitoneal injection of a mixture of ketamine hydrochloride (100 mg/kg body wt) and xylazine hydrochloride (5 mg/kg body wt). The level of anesthesia was maintained by hourly subcutaneous injections of one-third of the initial dose. MNTB single-unit recordings characteristically possess a prepotential, followed by a biphasic postsynaptic action potential, and in WT responded to sound from the contralateral ear (Kopp-Scheinpflug et al. 2003). The characteristic waveform allowed identification of spontaneous MNTB neuron firing even in the deaf mice. Spontaneous firing was recorded for a period of 4 s. Synaptic delay was measured from peak to peak between the prepotential and the postsynaptic action potential (Fig. 1*F*).

#### TTX experiments.

All measurements for the TTX experiments were carried out with tissue previously collected by Pasic and Rubel (Pasic et al. 1994; Pasic and Rubel 1991). These studies used adult Mongolian gerbils of either sex. Cochlear ablations were performed by removing the pinna, incising the tympanic membrane of one ear, and removing the malleus. The bony walls of all three turns of the cochlea were then opened, the cochlear contents were crushed and aspirated, and the modiolus was fractured. For TTX treatment, TTX crystals (Sigma Chemicals, St. Louis, MO) were suspended and placed on a disk of ethylene vinyl acetate copolymer resin (Elvax). Small pieces of the disk (0.1 g) containing ∼500 ng of TTX were cut with a 17-gauge stub adapter. TTX blockade of eighth nerve activity was obtained by making an incision posterior to the ear canal, opening the mastoid bulla, and placing the disk with TTX in the round window niche of the middle ear, resting against the round window membrane. In animals receiving TTX treatment for 48 h, the TTX disk was replaced after 24 h to ensure adequate maintenance of the block. Animals in the group that survived for 7 days had the disk containing TTX removed 20 or 44 h after insertion. Previous experiments showed that soma size of neurons in the cochlear nucleus is unaffected by placing polymer without TTX in the round window (Pasic and Rubel 1989) and that blockade reliably lasts for 4 h after removal of the disk (Pasic and Rubel 1991). All treatment was unilateral, and the MNTB contralateral to the treated ear was used for analysis. See Pasic and Rubel (1989, 1991) for complete methods.

#### Data analysis and statistical methods.

Statistical analyses of the data were performed with SigmaStat/SigmaPlot (SPSS Science, Chicago, IL) or Prism (GraphPad, La Jolla, CA). Results are reported as means ± SE; *n* = the number of animals for histological data and the number of neurons recorded from at least three different animals for electrophysiology data. Statistical comparisons between different data sets were made by unpaired Student's *t*-test or ANOVA. Differences were considered statistically significant at *P* < 0.05.

## RESULTS

### 

#### PMCA2 is involved in regulation of presynaptic transmitter release at calyx of Held.

Immunocytochemistry experiments demonstrated that PMCA2 is expressed throughout the MNTB. High-resolution images showing cross sections through a single MNTB neuron and the calyx of Held show that PMCA2 is present both presynaptically and postsynaptically (Fig. 1, *A* and *B*). The cross section through the calyx shows the inner and outer membrane of the calyx. PMCA2 is clearly present in the calyx, indicating that it is involved in presynaptic calcium clearance. PMCA2 is also present in the soma of postsynaptic neurons, where it is likely to be involved in postsynaptic calcium clearance or to be transported into the downstream synapses.

A presynaptic rather than postsynaptic action of PMCA2 was supported by the analysis of mEPSCs during in vitro whole cell patch-clamp recordings. The lack of presynaptic PMCA2 in the *dfw*^*2J*^/*dfw*^*2J*^ mice caused an increase in mEPSC frequency from 16.6 ± 6.0 Hz (*n* = 4) in WT to 38.1 ± 4.6 Hz (*n* = 4; *P* = 0.029) in the *dfw*^*2J*^/*dfw*^*2J*^, suggesting a presynaptic increase in transmitter release (Fig. 1*C*). The amplitude of the mEPSCs remained unaltered (WT: 55.3 ± 10.2 pA, *dfw*^*2J*^/*dfw*^*2J*^: 50.8 ± 13.8 pA; *P* = 0.791). Activation of the calyx of Held input via electric fiber stimulation at the midline showed an increase in α-amino-3-hydroxy-5-methyl-4-isoxazolepropionic acid receptor (AMPAR) conductance from 132.7 ± 16.0 nS (*n* = 18) in WT to 187.9 ± 14.5 nS (*n* = 17; *P* = 0.015) in *dfw*^*2J*^/*dfw*^*2J*^, while decay time constants were unchanged between genotypes (Fig. 1*D*). Extracellular recordings of single MNTB neurons in vivo in the *dfw*^*2J*^/*dfw*^*2J*^ mice revealed no sound-evoked activity while stimulating the contralateral ear with either pure tones or noise pulses up to 90 dB SPL (data not shown). However, the in vivo recordings allowed the acquisition of spontaneous firing rates that were significantly increased in the *dfw*^*2J*^/*dfw*^*2J*^ mice (81.9 ± 21.70 Hz; *n* = 13) compared with their WT controls (25.7 ± 4.0 Hz; *n* = 65, *P* = 0.001; Fig. 1*E*). The large somatic calyx synapses that innervate each MNTB neuron give rise to a typical complex waveform from in vivo extracellular recordings (Guinan and Li 1990; Kopp-Scheinpflug et al. 2003) consisting of a presynaptic potential (prepotential) and a postsynaptic action potential (recording traces in Fig. 1*F*). The prepotential and the postsynaptic action potential are separated by a synaptic delay, which was shorter in the *dfw*^*2J*^/*dfw*^*2J*^ mice (0.38 ± 0.01 ms; *n* = 13) compared with WT (WT: 0.47 ± 0.02 ms; *n* = 14, *P* = 0.002; Fig. 1*F*).

**Fig. 2.**
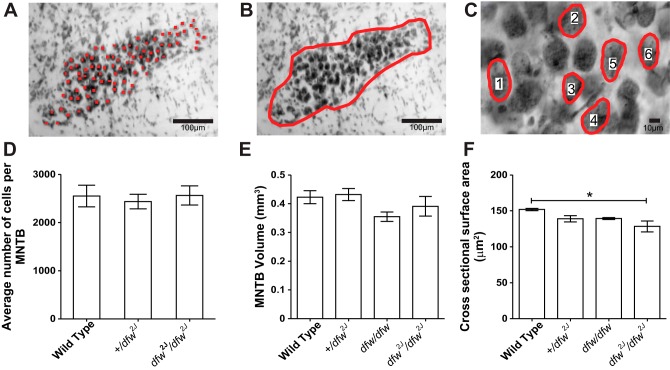
MNTB morphology is similar in wild type and *dfw*^*2J*^ mutants. *A–C*: Nissl-stained coronal sections were used to estimate cell number (*A*), MNTB volume (*B*), and average cell size (*C*) in wild type and +/*dfw2J* and *dfw*^*2J*^/*dfw*^*2J*^ mutants. Red circles and numbers in C indicate examples of measured MNTB cells. *D*: there was no significant difference in cell number between any of the genotypes (*n* = 3 mice/group). *E*: there was no significant difference in MNTB volume between any of the genotypes (*n* = 6 MNTBs/group). *F*: there was a significant decrease in the average cross-sectional area between wild type and *dfw*^*2J*^/*dfw*^*2J*^ mutants (*P ≤ 0.05, *n* = 1,854 cells from 9 mice). Error bars show SE.

Together these data support the hypothesis that PMCA2 is involved in the regulation of presynaptic transmitter release at the calyx of Held. To test whether PMCA2 is necessary for neuronal survival or normal neuronal morphology, MNTB neuron number (Fig. 2*A*), nucleus volume (Fig. 2*B*), and neuron size (Fig. 2*C*) were measured in Nissl-stained sections from +/+ littermates and +/*dfw*^*2J*^ and *dfw*^*2J*^/*dfw*^*2J*^ mice. Each MNTB contained on average 2,551 (WT), 2,436 (+/*dfw*^*2J*^), and 2,563 (*dfw*^*2J*^/*dfw*^*2J*^) neurons. Average MNTB volumes were 0.42 mm^3^, 0.43 mm^3^, 0.35 mm^3^, and 0.39 mm^3^ in WT, +/*dfw*^*2J*^, *dfw*/*dfw*, and *dfw*^*2J*^/*dfw*^*2J*^ mice, respectively. Statistical analysis confirmed that there was no significant difference in neuron number (Fig. 2, *A* and *D*; *F* = 0.1310; *P* = 0.8797) or in the volume of the MNTB nucleus (Fig. 2, *B* and *E*; *F* = 1.965; *P* = 0.4508) between the genotypes.

#### Cell size gradient discovered in wild-type mice is absent in PMCA2 mutants (deafwaddler mice).

**Fig. 3.**
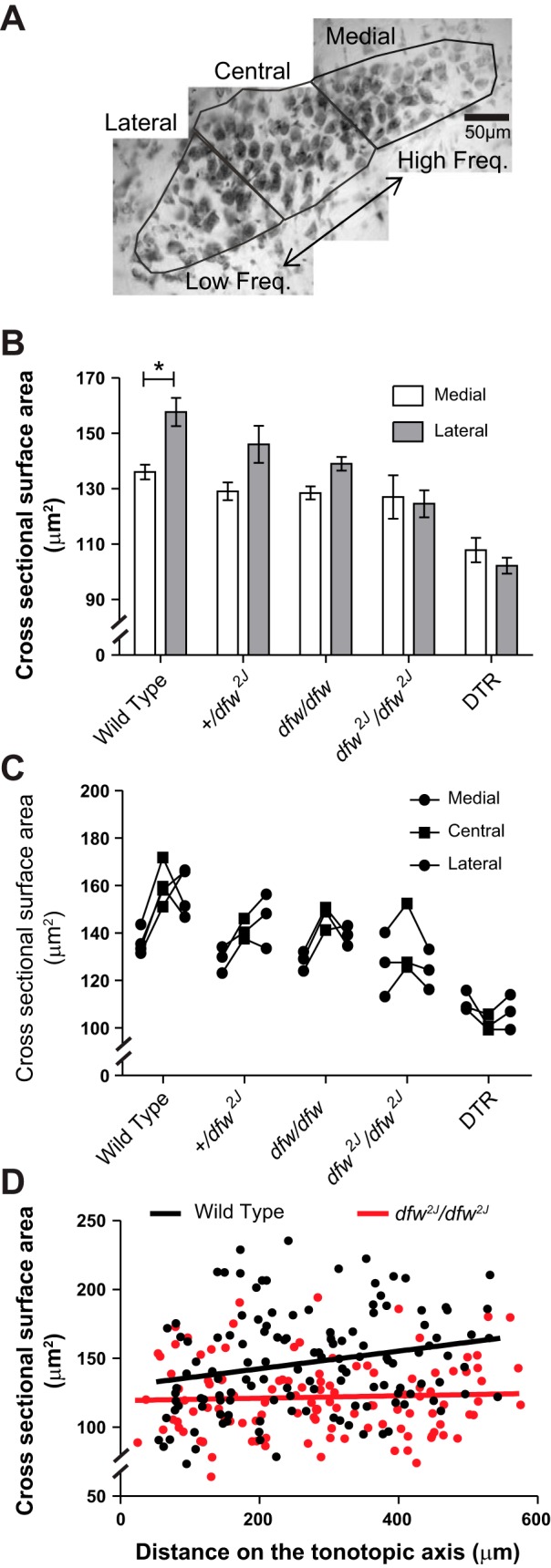
Medial-to-lateral soma size gradient in the MNTB is absent in *dfw*^*2J*^ mutants. *A*: cells were defined as medial if located in the medial third of the MNTB or lateral if located in the lateral third of the MNTB. *B*: there was a significant increase in the size of lateral cells compared with medial cells in the wild-type animals (*P ≤ 0.001). There was no significant increase in the size of lateral cells in +/*dfw*^*2J*^, *dfw*/*dfw*, *dfw*^*2J*^/*dfw*^*2J*^, or DTR mice. Error bars show SE. *C*: individual average cell size for medial and lateral cells in each MNTB. *D*: scatterplot of location along the tonotopic axis vs. cross-sectional surface area for 1 MNTB from a wild-type and a *dfw*^*2J*^/*dfw*^*2J*^ mouse. The linear regression is significantly different from zero for wild type (*P* = 0.01) but not for *dfw*^*2J*^/*dfw*^*2J*^.

MNTB neurons were significantly smaller in *dfw*^*2J*^/*dfw*^*2J*^ (128.36 ± 7.54 μm^2^) than in WT (151.89 ± 1.11 μm^2^; Fig. 2, *C* and *F*; *F* = 5.894; *P* = 0.04). To determine whether these differences showed any tonotopic relationship, the nucleus was divided into thirds and neurons were assigned to medial, central, and lateral groups (Fig. 3*A*). We defined PMCA2 function as the percentage of PMCA2 protein, determined by the number of functional alleles possessed by an animal, multiplied by the PMCA2 pumping efficiency, determined by biochemical assay (Penheiter et al. 2001) and compared to WT. We tested a range of PMCA2 function from WT (which have 100% protein), +/*dfw*^*2J*^ with ∼50% protein, *dfw*/*dfw* with ∼30% function as measured by a calcium clearance assay (Penheiter et al. 2001), and *dfw*^*2J*^/*dfw*^*2J*^, which have no functional PCMA2 protein (Table 1). In WT animals, medial neurons were significantly smaller (136.01 ± 2.66 μm^2^) than lateral neurons (157.71 ± 5.05 μm^2^; Fig. 3*B*; *P* = 0.02). In +/*dfw*^*2J*^, the location-dependent difference in neuronal cell size was decreased and no longer significant (Fig. 3*B*; *P* = 0.08). The size difference was decreased further in *dfw*/*dfw* and was absent in the *dfw*^*2J*^/*dfw*^*2J*^ mice (Fig. 3*B*). Although absolute neuronal soma size varied slightly between animals, comparing the size difference in medial and lateral neurons for each individual mouse confirmed the presence or absence of the overall size gradient in the different genotypes (Fig. 3*C*). Neuronal soma size data of all measured individual neurons from one WT MNTB and one *dfw*^*2J*^/*dfw*^*2J*^ MNTB are shown as an example in Fig. 3*D*. The slope of the linear regression for neuronal soma size is significantly nonzero in WT mice (Fig. 3*D*; *P* = 0.01), while no relationship between neuronal soma size and tonotopic location was found in *dfw*^*2J*^/*dfw*^*2J*^, demonstrating that there is a neuronal cell size gradient in WT that is absent in *dfw*^*2J*^/*dfw*^*2J*^.

**Table 1.** Comparison of PMCA2 function in deafwaddler mutants and DTR mice

Genotype	Hearing Phenotype	PMCA Protein	PMCA Efficiency	PMCA Function
Wild type	Normal	100%	100%	100%
*+*/*dfw*^*2J*^	High-frequency loss	∼50%	100%	∼50%
*dfw*/*dfw*	Deaf	100%	∼30%	∼30%
*dfw*^*2J*^/*dfw*^*2J*^	Deaf	0%	0%	0%
DTR	Deaf	100%	100%	100%

The % of PMCA protein is calculated based on the number of functional alleles possessed by an animal, assuming that all alleles produce the same amount of protein. Because of the premature stop codon in the *dfw2J* mutation, no protein is produced. The efficiency of PMCA2 was calculated by Penheiter and colleagues with a calcium clearance assay in the *dfw* mutants (Penheiter et al. 2001). In +/*dfw2J* heterozygotes the efficiency of the existing PMCA will be wild type like, but its overall function in the animal only amounts to 50%.

#### Medial-to-lateral increase in membrane capacitance is accompanied by larger synaptic input in wild type but not in dfw^2J^ mutants.

**Fig. 4.**
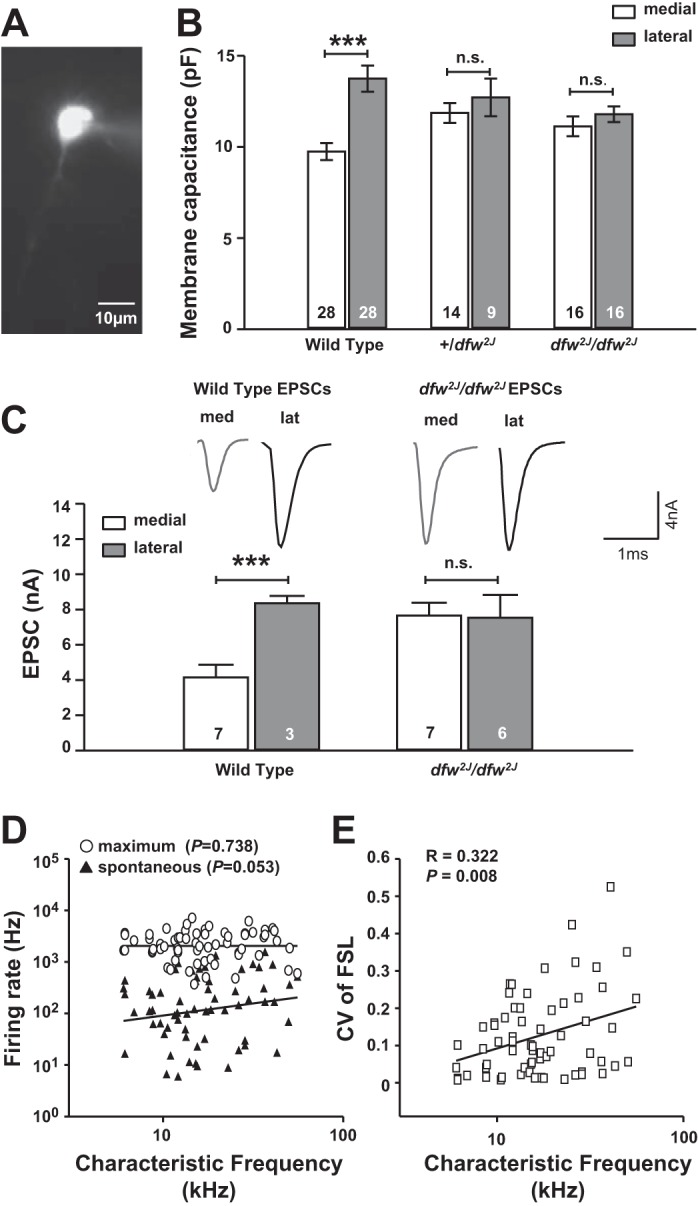
Lateral MNTB neurons have a larger membrane capacitance and larger calyceal inputs in wild type but not in *dfw*^*2J*^ mutants. *A*: cell membrane capacitance was acquired for visually identified neurons in voltage-clamp mode. Dye labeling of each neuron via the patch pipette allowed off-line measurements of the neurons' position within the MNTB. *B*: capacitance measurements corroborate histology data showing that lateral cells are significantly larger than medial cells in wild type (*P* ≤ 0.001) but there is no significant difference between cells in +/*dfw*^*2J*^ and *dfw*^*2J*^/*dfw*^*2J*^ mutants (n.s.). Error bars show SE. *C*: calyceal EPSCs are larger in lateral than in medial MNTB wild-type neurons. No significant difference was found between EPSC amplitudes of medial and lateral neurons in *dfw*^*2J*^/*dfw*^*2J*^ mutants. *D*: in in vivo recordings of single MNTB neurons in wild types, characteristic frequency is used as a measure for medial-to-lateral position. No significant correlation was found between medial-to-lateral position and firing rate. *E*: the coefficient of variation for the first spike latency (FSL) showed a positive correlation with characteristic frequency. Unfortunately, because of the deafness phenotype these data could not be acquired in the *dfw*^*2J*^/*dfw*^*2J*^ mutants. ***P ≤ 0.001, n.s., not significant.

As a complementary measure of neuronal soma size, somatic surface area was assessed by determining the cell membrane capacitance (*C*_m_) in voltage-clamp recordings of MNTB neurons and comparing it across the tonotopic axis (see materials and methods). In WT mice, medial MNTB neurons had a smaller capacitance (*C*_m_: 9.75 ± 2.47 pF; *n* = 28) than lateral MNTB neurons (*C*_m_: 13.75 ± 0.72 pF; *n* = 28; Fig. 4*B*; *P* = 0.001), corroborating the size gradient measured in the histological experiments. The difference in *C*_m_ between medial and lateral neurons was completely abolished in +/*dfw*^*2J*^ mice [medial *C*_m_: 11.87 ± 0.55 pF (*n* = 14); lateral *C*_m_: 12.72 ± 1.03 pF (*n* = 9); *P* = 0.463] as well as in *dfw*^*2J*^/*dfw*^*2J*^ mice [medial *C*_m_: 11.14 ± 0.54 pF (*n* = 16); lateral *C*_m_: 11.79 ± 0.42 pF (*n* = 16); *P* = 0.344]. When compared across genotypes the differences in capacitance between medial neurons or lateral neurons were not significantly different (repeated-measures ANOVA). No systematic changes in input resistance between medially and laterally patched cells in the MNTB of WT, +/*dfw*^*2J*^, or *dfw*^*2J*^/*dfw*^*2J*^ mice were observed (repeated-measures ANOVA: *P* = 0.257). In contrast, membrane time constants (τ) in WT MNTB were significantly faster in medial (7.4 ± 0.7 ms; *n* = 16) than in lateral (10.8 ± 1.0 ms; *n* = 16; *P* = 0.007) neurons, while no such correlation was found in the +/*dfw*^*2J*^ or *dfw*^*2J*^/*dfw*^*2J*^ mice (data not shown).

The difference in soma size between medial and lateral MNTB neurons raised the question of whether the synaptic current or the neuronal output firing also varied across the tonotopic axis. Our initial experiments (Fig. 1) comparing overall EPSCs between WT and *dfw*^*2J*^/*dfw*^*2J*^ mice already showed larger EPSCs in the *dfw*^*2J*^/*dfw*^*2J*^ mice. Sorting the EPSCs according to the location of the neurons within the MNTB revealed significantly larger EPSCs in lateral, low-frequency MNTB neurons (8.4 ± 0.4 nA; *n* = 3) than in medial, high-frequency neurons (4.2 ± 0.7 nA; *n* = 7, *P* = 0.007; Fig. 4*C*). In the *dfw*^*2J*^/*dfw*^*2J*^ mice calyceal inputs to medial and lateral neurons were equally large (medial: 7.7 ± 0.7 nA; *n* = 7; lateral: 7.5 ± 1.3 nA; *n* = 6; *P* = 0.931). Larger EPSCs in lateral, low-frequency MNTB neurons could affect either firing rates or temporal precision or both. In vivo recordings in WT MNTB neurons showed no significant correlation of characteristic frequency (i.e., location along the medial-to-lateral axis) with either spontaneous (Pearson correlation: *P* = 0.53) or maximum (Pearson correlation: *P* = 0.73; Fig. 4*D*) firing rates. In contrast, a significant correlation (Pearson correlation: *P* = 0.008) between the coefficient of variation of the first spike latency to sound-evoked responses and the characteristic frequency was found in WT mice (Fig. 4*D*). The deafness phenotype of the *dfw*^*2J*^/*dfw*^*2J*^ mice did not permit a similar analysis in the mutant.

#### Lack of auditory activity reversibly eliminates neuronal cell size gradient in MNTB.

**Fig. 5.**
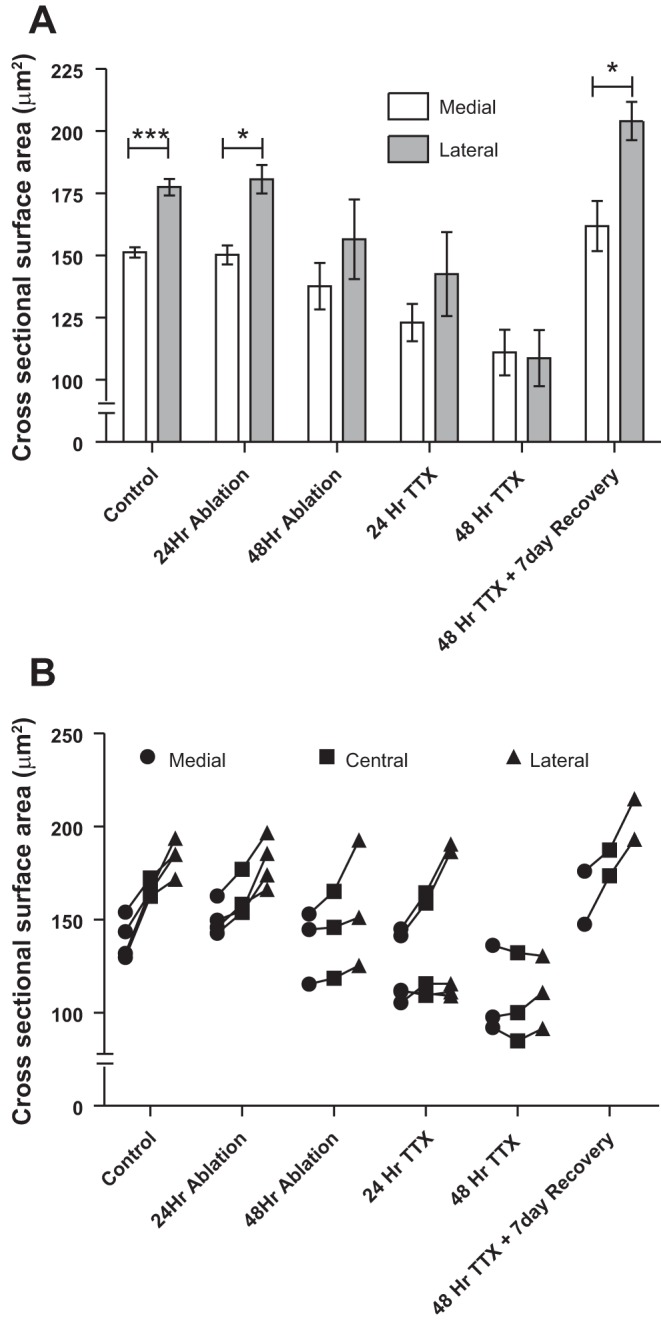
Lack of sensory input reversibly abolishes the soma size gradient in gerbils. *A*: control gerbils showed a cell size gradient, as did subjects with tissue collected 24 h after cochlear ablation (***P ≤ 0.001 and *P = 0.01, respectively). Tissue collected 48 h after cochlear ablation showed a diminished gradient. Gerbils treated with TTX for 24 or 48 h showed a decreased or no cell size gradient, but in those animals allowed to recover for 7 days, the gradient had returned (*P = 0.02). Error bars show SE. *B*: individual average cell size for medial, central, and lateral regions of each MNTB. ***P ≤ 0.001, *P ≤ 0.05.

In vivo recordings of MNTB neurons revealed that the *dfw*^*2J*^/*dfw*^*2J*^ mice had no measurable responses to sound but maintained spontaneous action potential firing activity (Fig. 1*F*), which is known to be generated in and propagated from the cochlea (Lippe 1994; Tritsch et al. 2010). To determine whether the elimination of cochlear activity could also cause a change in the neuronal cell size gradient, we used three different approaches (Table 1): First, we eliminated all cochlear hair cells by administering DT to mice that selectively express the human DTR in their hair cells (Tong et al., 2015). These mice showed an overall decrease in MNTB neuronal cell size by ∼30% compared with WT and no significant difference in size between medial and lateral neurons (Fig. 3, *B* and *C*). Second, we used tissue from animals either 24 or 48 h after cochlear ablation. These experiments were performed in gerbils, which are slightly larger than mice; this simplifies the surgery and at the same time allows a generalization of the activity-dependent neuronal cell size gradient to a mammal that hears in the lower frequency range. Similar to the data from WT mice, we found that medial neurons (151.26 ± 2.03 μm^2^) in the gerbil MNTB are significantly smaller than lateral neurons (177.51 ± 3.32 μm^2^; Fig. 5; *P* ≤ 0.001). Twenty-four hours after cochlear ablation the difference in size between medial and lateral neurons was still significant (Fig. 5*A*; *P* = 0.01), but 48 h after cochlear ablation the difference was no longer significant, indicating that the size gradient had decayed (Fig. 5). The third approach to eliminate cochlear activity asked whether the loss of the neuronal soma size gradient following sensory deprivation was reversible. The sodium channel blocker TTX, which prevents the generation of action potentials in the spiral ganglion cells and therefore eliminates all cochlear driven activity, was applied via the round window (see materials and methods). After 24 h of TTX treatment the size difference between medial and lateral neurons was no longer significant, and by 48 h the soma size was indistinguishable between medial and lateral cells (Fig. 5). Data for the average sizes for medial, central, and lateral neurons are shown for each individual gerbil in Fig. 5*B*.

The pharmacological blockade of sodium channels by TTX was reversible, so that after TTX was removed the cochlea recovered and activity resumed. In animals that were allowed to recover for 7 days from a 48-h TTX treatment, the size gradient was restored and lateral neurons were again larger than medial neurons (Fig. 5; *P* = 0.02).

## DISCUSSION

The results of this study show a medial-to-lateral cell size gradient in the MNTB. This gradient is dependent on afferent activity and can be reversibly abolished when the input activity is lost. While TTX and DT treatment or cochlear ablation completely eliminates all input activity, the *deafwaddler* mutation maintains spontaneous firing but cannot transmit additional sound-evoked activity. All of these manipulations led to smaller cells. If there was a simple or linear correlation between firing rate and cell size, then we would have predicted a uniformly large cell size in the *dfw*^*2J*^ mutants, given the high spontaneous firing rates of the mutant mice. However, general afferent activity (spontaneous firing) alone did not lead to larger lateral neurons. Therefore our observations suggest a more complex control of soma size, perhaps including the release of calcium-dependent signals controlling the size of the lateral neurons. Sound-frequency specific input characteristics seem necessary to induce and maintain the neuronal size gradient, and PMCA2 is involved in regulating these inputs.

### 

#### Tonotopic gradients in the auditory system.

**Fig. 6.**
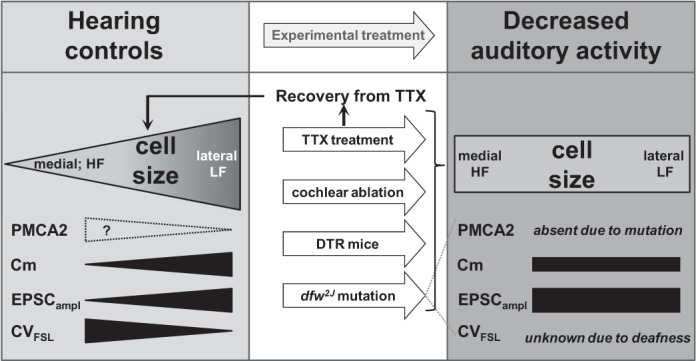
Sound-evoked auditory activity is required to maintain the soma size gradient in the MNTB of mice and gerbils. Normal-hearing gerbils and mice both show a soma size gradient. If auditory activity is eliminated through cochlear ablation (gerbil) or TTX treatment (gerbil), DT treatment (mouse), or genetic mutation (mouse) the cell size gradient is absent. If auditory activity returns after a period of deprivation (TTX treatment) the cell size gradient will be restored. The dotted outline of the presumptive PMCA2 gradient indicates expression in the calyces as suggested by the electrophysiological data rather than in MNTB neurons. HF, high frequency; LF, low frequency.

Tonotopic organization is first established in the cochlea, where the location of hair cells along the basilar membrane dictates the characteristic frequency to which the hair cells respond through both physical resonance and molecular signaling mechanisms. This organization is propagated to many higher levels of the auditory brain stem and all the way to the auditory cortex. Tonotopic gradients in cell morphology and size as well as gradients involving ion channels and receptors are well established for many different parts of the auditory pathway: The hair cells in the cochlea show differences in stereocilium length and somata size. Apical cells, responding best to low frequencies, have longer stereocilia and larger somata, while basal cells, responding to high sound frequencies, have shorter stereocilia and smaller somata (Ashmore and Gale 2000; Corwin and Warchol 1991; Tilney et al. 1987). For example, in the spiral ganglia there is a tonotopic arrangement of synaptic proteins associated with greater expression of α-GluR2/3 in high-frequency neurons than in low-frequency neurons (Flores-Otero and Davis 2011). In the MNTB ion channel gradients of K_v_3 decrease across the medial-to-lateral tonotopic axis (Leao et al. 2006; von Hehn et al. 2004) while an inverse K_v_1 gradient increases from medial to lateral MNTB (Gazula et al. 2010; Leao et al. 2006). These tonotopic gradients have been recognized throughout the developing and mature auditory pathways (Rubel 1978; Smith and Rubel 1979) and are considered essential features for each neuron to optimally perform specialized tasks (within the context of achieving temporal precision and information transmission across a range of firing). In this study we have characterized a cell size gradient in the MNTB that is dependent on auditory activity. As summarized in Fig. 6, four independent approaches were employed to test whether maintenance of the gradient requires active auditory inputs. Two methods utilized mouse transgenic mutants, and two used surgical and pharmacological manipulation of the cochlea in gerbils. The TTX treatment in gerbils provided a reversible procedure that demonstrated that the neuronal size gradient in the MNTB is able to recover after a period of sensory deprivation. A previous publication noted a difference in MNTB cell size between medial and lateral cells (Pasic and Rubel 1991). However, at that time we were unable to relate a continuous gradient to the tonotopic organization of MNTB. Previous reports have eliminated all cochlea-driven activity (both sound evoked and spontaneous), but the *dfw*^*2J*^/*dfw*^*2J*^ model used in the present study allowed distinction between the influence of spontaneous and sound-evoked activity. Mutant *dfw*^*2J*^/*dfw*^*2J*^ mice are deaf (Street et al. 1998), and no acoustically driven activity could be recorded in the MNTB of these mice. However, high levels of spontaneous activity are maintained and were recorded in the MNTB of *dfw*^*2J*^/*dfw*^*2J*^ mice. Further investigations will be required to determine whether the size gradient develops if either high- or low-frequency input is eliminated before hearing onset.

#### How PMCA2 could affect gradients of MNTB function along medial-to-lateral axis.

Knowledge of PMCA2 expression along the medial-to-lateral axis in the MNTB would provide insight into how PMCA2 could influence MNTB function. However, immunohistochemical labeling is difficult to quantify, and the MNTB is too small to provide sufficient protein for Western blot analysis of medial and lateral divisions, especially given that this method could also not distinguish between calyceal and somatic PMCA2. Therefore in the present study we used physiological parameters to test postulates concerning PMCA2 expression within the MNTB. Lateral neurons of WT animals have larger EPSC amplitudes that in vivo can cause either a higher MNTB firing rate or higher temporal precision or both. We plotted the in vivo firing rates against the tonotopic (medial to lateral) axis and found no significant correlation. In contrast, plotting the coefficient of variation of the first spike latency (as a measure of temporal precision) against the tonotopic axis showed low coefficients of variation in the low-frequency (lateral) MNTB neurons. Unfortunately, the deafness phenotype of the *dfw*^*2J*^*/dfw*^*2J*^ mice did not allow a similar analysis in the mutant, but the WT data corroborate the idea that low-frequency (lateral) calyx synapses express less PMCA2, which results in less suppression, larger EPSCs, and well-timed action potentials in the low-frequency neurons. Rather than arguing for an “increased” EPSC amplitude in medial MNTB of *dfw*^*2J*^ mice, we interpret this result as less suppression of the EPSCs, compared with their WT counterparts. The amplitude of the synaptic response strongly depends on basal and dynamic presynaptic calcium concentrations in the terminal (Billups and Forsythe 2002; Bollmann et al. 2000; Kochubey et al. 2009). PMCA2 in the WT calyx of Held contributes to calcium clearance from the terminal, while in the *dfw*^*2J*^ mutant the lack of PMCA2 in the calyx of Held slows calcium extrusion rates and raises basal intracellular calcium concentrations, creating a complex interaction with multiple mechanisms of short-term plasticity (Muller et al. 2010) and causing increased transmitter release. Applying similar logic to the differences in EPSC size between medial and lateral MNTB neurons in the WT leads to the conclusion that PMCA2 is more highly expressed in the medial MNTB and this causes the smaller EPSC amplitudes in medial neurons. Such a distribution of PMCA2 in vivo causes larger EPSCs with shorter synaptic delay and less timing jitter in lateral neurons. Higher PMCA2 expression in medial neurons would increase calcium clearance, causing EPSCs with chronically depressed amplitudes, which are sensitive to recent history but poorly timed (Lorteije et al. 2009).

The lack of PMCA2 in both the medial and the lateral MNTB neurons in the *dfw*^*2J*^ could be interpreted as medial *dfw*^*2J*^ neurons lacking the chronic depression present in WT (thus generating larger EPSC amplitudes in the mutant). Although it is not our intention to exclude a peripheral component to the net changes in auditory processing induced by the loss of PCMA2, the larger amplitude of the calyx of Held EPSC in the mutant mice strongly supports a local and central mechanism of action, since each EPSC is generated by the action of a single synaptic input (the calyx), which has arisen from the globular bushy cell in the anterior ventral cochlear nucleus. Similar effects (increased EPSCs) in the periphery (at the hair cell or endbulb) might increase the frequency of action potential firing in the bushy cell axons but would not directly influence the amplitude of evoked synaptic currents at the calyx. This interpretation is consistent with previous reports that the deafness phenotype of the *dfw* mutant arises in the cochlear hair cells as initially described (Street et al. 1998), while we conclude that the central expression of PMCA2 further affects transmitter release and neuronal cell size in the auditory brain stem (see below).

#### Balance between input size and cell size.

EPSC frequency and size are influenced by the available calcium in the presysnaptic terminal. Eliminating PMCA2 from the calyx of Held terminal will raise presynaptic calcium concentrations, increasing transmitter release and causing larger EPSCs, whereas in WT MNTB PMCA2 will maintain lower basal intracellular calcium concentrations, and thereby fine-tune synaptic strength (Billups and Forsythe 2002; Borst et al. 1995; Felmy and Schneggenburger 2004; Felmy and von Gersdorff 2006).

*C*_m_ is proportional to the surface area of a cell, and higher capacitance slows the neuronal membrane time constant: τ = *R*_m_ × *C*_m_ (where *R*_m_ is the resistance of the membrane), so that smaller neurons will fire more rapidly than large neurons (Franzen et al. 2015). The soma size gradient in the MNTB implies that medial cells (which are smaller than lateral cells) will fire more rapidly than lateral cells. However, there are other demands on neurons; for example, one reason for larger cell bodies in the lateral, low-frequency region of the MNTB might be a higher metabolic rate in these neurons. High metabolic rate is often associated with larger cells, and it has been suggested that neurons that process signals with a high temporal resolution have especially high metabolic demands (Attwell and Laughlin 2001). The present results suggest the possibility of a homeostatic adjustment in which larger synaptic inputs, which enable high temporal precision of the lateral MNTB neurons, are complemented by larger postsynaptic cells and suggestive of higher metabolic demand.

Consequently, not only the cell size but also increasing the rate or amplitude of the synaptic inputs increases the energy demands of the cell (Sengupta et al. 2013). We conclude that PMCA2 expression in these giant synapses innervating medial MNTB neurons causes synaptic suppression (compared with their lateral counterparts). This might reduce the energy demand of the medial neurons and trigger a reduction in neuronal size. Further work will be necessary to test these hypotheses.

## GRANTS

This research was funded by National Institute on Deafness and Other Communication Disorders Auditory Neuroscience Training Grant DC-005361 (J. H. Weatherstone), RO1 DC-02739 (B. L. Tempel), P30Core DC-04661 (E. W. Tempel), and R01 DC-03829 (E. W. Tempel), Deutsche Forschungsgemeinschaft SFB870/2-A10 (C. Kopp-Scheinpflug), and Medical Research Council K005170 (I. D. Forsythe).

## DISCLOSURES

No conflicts of interest, financial or otherwise, are declared by the author(s).

## ENDNOTE

At the request of the authors, readers are herein alerted to the fact that additional materials related to this manuscript may be found at the institutional website of one of the authors, which at the time of publication they indicate is: http://depts.washington.edu/tempelab/Protocols/DFW2J.html. These materials are not a part of this manuscript, and have not undergone peer review by the American Physiological Society (APS). APS and the journal editors take no responsibility for these materials, for the website address, or for any links to or from it.

## AUTHOR CONTRIBUTIONS

J.H.W., C.K.-S., N.P., and Y.W. performed experiments; J.H.W., C.K.-S., and N.P. analyzed data; J.H.W., C.K.-S., I.D.F., E.W.R., and B.L.T. interpreted results of experiments; J.H.W. and C.K.-S. prepared figures; J.H.W., C.K.-S., and B.L.T. drafted manuscript; J.H.W., C.K.-S., N.P., Y.W., I.D.F., E.W.R., and B.L.T. edited and revised manuscript; J.H.W., C.K.-S., N.P., Y.W., I.D.F., E.W.R., and B.L.T. approved final version of manuscript.
